# Mental health status and coping strategies of Chinese university students during the COVID-19 pandemic: A rapid review

**DOI:** 10.1371/journal.pone.0296309

**Published:** 2023-12-22

**Authors:** Wenjie Luo, Jalal Mohammed

**Affiliations:** 1 Department of Pharmacy, Xiangyang No.1 People’s Hospital, Hubei University of Medicine, Shiyan, China; 2 Faculty of Health and Environmental Sciences, Auckland University of Technology, Auckland, New Zealand; 3 Faculty of Health, University of Canterbury, Christchurch, New Zealand; Kwame Nkrumah University of Science and Technology, GHANA

## Abstract

Since the coronavirus (COVID-19) outbreak in December 2019, students have been under unparalleled psychological stress worldwide. As part of its prevention and control strategies, the Chinese Ministry of Education proposed online teaching activities for universities. For the first time, teaching and learning shifted completely online, significantly impacting university students used to classroom learning. This research addresses the knowledge gap about the mental health and coping strategies employed by Chinese university students during the COVID-19 pandemic. Electronic databases (PsycINFO, Scopus, Medline, Cochranes and CNKI) were searched systematically from 2019 to 2023, as part of this literature review. From the 349 articles found, 25 met the inclusion criteria for analysis. Thematic analysis was used to identify six sub-themes, organized under two main themes: Mental health issues of Chinese university students and their coping mechanisms. Heightened stress, anxiety, and depression appeared in Chinese university students during the pandemic, which may have been compounded by their isolation and the disruptions to their studies. Although the impact of COVID-19 on Chinese university students is waning, this study emphasizes the potential long-lasting impact on their mental health, which requires further investigation, particularly regarding gender differences. Moreover, positive and negative coping strategies were found in this review. Strategies for seeking social and family support and participating in sports activities had significant alleviating effects, while negative coping strategies such as alcohol-use and smoking did not. This rapid review informs the development of policies and interventions to enhance the mental health of university students during crisis events. The findings serve to inform health policymakers, university psychologists, and educators in improving the well-being of this student population.

## Introduction

Pandemics have disrupted social order and affected public health throughout human history [1]. The coronavirus (COVID-19) has rapidly spread across China and other nations since it was identified in Wuhan [2, 3]. Subsequently, a significant public health emergency of international concern (PHEIC) has been triggered by COVID-19, posing a threat to human life and mental health [3, 4]. As of November 2022, 634 million cases of coronavirus and 6.6 million deaths have been reported worldwide [5]. As the impacts of the pandemic grow, health systems in many countries have been overwhelmed, and health care for millions of people has been delayed [6]. Government authorities are now turning their attention from mitigating the spread to managing long-term impacts and implications for health systems [7, 8].

During the height of the outbreak, most people altered their daily routines and spent less time interacting with others to prevent the spread of coronavirus, prompted and reminded by public health messaging. This dearth of information has repeatedly stimulated people, with an increasing number of people reporting emotional irregularities and cognitive imbalances that have affected their ability to pay attention and remember what they have read [9]. There has also been a rise in reports of changes in behavior and physical symptoms such as nausea, vomiting, and diarrhea [10]. Overreactions can amplify the emotional toll and pose health risks physically and mentally [11, 12]. As a result, mental health status can be affected and, in cases, worsened.

### Prevention and control measures

China implemented a wide range of prevention and control measures to eliminate the spread of COVID-19 (see Table 1). In response, Chinese education institutions acted swiftly during the COVID-19 outbreak, implementing various precautions to protect their students’ safety and health [13]. As one of the earliest and hardest hit countries, Chinese universities were at the forefront of efforts to control the pandemic. Many universities implemented online learning to replace in-person classes to respond to the outbreak. They expected such methods to help control the coronavirus spread between students, teachers, and the wider population [14]. Students and faculty members would have otherwise been at a higher risk of contracting the virus through close contact in crowded classrooms and lecture halls. Additionally, Chinese universities implemented strict entry and exit controls, such as temperature checks and health screening questionnaires, to identify potential cases and prevent infected individuals from entering campus [15]. These measures were crucial in identifying potential cases early on and preventing spread within the university community.

**Table 1:** Prevention and control measures for COVID-19 pandemic in China.

Date	Prevention Measures	Details
January 2020	Lockdown	A strict lockdown was imposed in Wuhan and other cities in Hubei Province. This has effectively quarantined tens of millions of people and helped to control the spread of the COVID-19.
February 2020	Wartime Strategy	This included measures such as ramping up production of medical supplies and equipment, building new hospitals, and mobilizing medical personnel from across the country to Wuhan.
March 2020	(1) COVID-19 Test (2) Contact Tracing (3) Travel Restrictions	(1) Chinese government ramped up testing for COVID-19 and announced that it would test all 11 million residents of Wuhan. (2) Tracing people who have had contact with individuals who have tested positive for COVID-19 through health code systems or digital data. (3) Isolating individuals coming from high-risk areas and restricting travel between different regions within the country.
December 2021	"Dynamic Zero COVID-19"	(1) Severe restrictions on the movement of people (2) Large-scale nucleic acid detection (3) Fast tracking and isolation (4) Promoting vaccination (5) Strengthening health protection
February 2023	End of COVID-19 pandemic in China	Prevention and control measures for Class B infectious diseases

Source: National Health Commission of the People’s Republic of China [17].

To further reduce the risk of transmission, many universities required students, faculty, and staff who had travelled to areas with high numbers of COVID-19 cases or who had come into contact with infected individuals to quarantine for 14 days before returning to campus [16]. This strategy ensured that those who may have been introduced to the virus could be isolated and, if necessary, receive medical care reducing the risk of further transmission within the university community. These control measures were instrumental in preventing the spread of the virus on campus and protecting students, faculty, and staff. However, the stringent measures and COVID-19 altered the normal academic life of Chinese university students, which impacted their mental health.

### Mental health context

According to UNESCO [18], national lockdowns have impacted around 60% of students worldwide. The transition to online instruction due to COVID-19 lockdown measures adversely affected students’ mental health well-being, as shown by numerous studies [19]. Amerio found that social isolation during the lockdown negatively affected students’ mental health [9]. Adding to this understanding, Patelarou found that approximately one-third of nursing students in Spain, Greece, and Albania experienced moderate depression during the pandemic [20]. Interestingly, students in Spain had a significantly higher impact (59.1%) followed by Albania (34.5%) and Greece (21.8%). This might be attributed to the increasing number of COVID-19 cases in the region, which led to heightened public concern and anxiety. Similarly, a survey of university students in Bangladesh reported increased levels of agitation (28.5%), anxiety (33.3%), and depression (46.9%) during the pandemic [21]. In Japan, Ueda noted that the economic consequences of the pandemic, along with the direct health effects of COVID-19, impacted the mental well-being of university students [22]. Likewise, Lee found that students in Daegu, South Korea, experienced higher levels of depression and anxiety during the pandemic [23]. Similar finding across the global student community indicate that lockdown measures have had negative impacts on students’ mental health and suggest that interventions specific to this group are needed.

As the coronavirus spread, Chen found that a majority of Chinese students were at high or moderate risk of stress management, psychological state, risk perception, behavioural patterns, family relationships, academic stress, and peer relationships [15]. This was illustrated in particular among Chinese medical students, who experienced an increase in anxiety, melancholy, and sleep disturbances [24]. The prevalence of post-traumatic stress disorder (PTSD) in Wuhan, the region most affected by the pandemic, was 7% [25]. Notably, there were differences on the impact on mental health, particularly regional variations, with some regions reporting a greater impact on mental health. This can be seen in the Guangdong province where the rate of depression was 21.2%, and the rate of anxiety was 26.6%, both significantly higher than the national rate for students [26]. Furthermore, Gao found that depression and anxiety levels were higher than pre-covid levels [27]. This corresponds to findings that students may experience mental health issues when exposed to severe public health events.

## Methodology

### Data sources and search strategy

This rapid review used the PICO framework (population, intervention, comparison and outcome,) to guide the literature search process [28, 29]. The PICO framework was used to focus the search and define search terms. Examples of search terms used to locate literature on the psychological well-being and coping strategies of Chinese university students include: ‘China’ or ‘Chinese*’ or ‘Asia’ and ’mental health’ or ‘emotional’ or ‘psychological*’ and ‘university students’ or ‘postgraduate’ or ‘undergraduate*’ or ‘college students’ and ‘adaptation’ or ‘(coping strategies)*’ or ‘coping skills’ and ‘COVID-19*’ or ‘2019 nCov infection’ or ‘SARS- Cov-2’ or ‘coronavirus disease-19’. Literature search results were restricted to peer-reviewed articles, with the year of publication limited from 2019 to 2023. During the search process, an asterisk (*) symbol after a keyword was used as truncations to facilitate the capture variations of the term. In addition, Boolean operators such as "or" and "and" were used to obtain an exact search range, including articles matching any search terms. As the target group of this study was Chinese university students, both Chinese and English language articles were included in the review.

### Data analysis and synthesis

**Fig 1:**
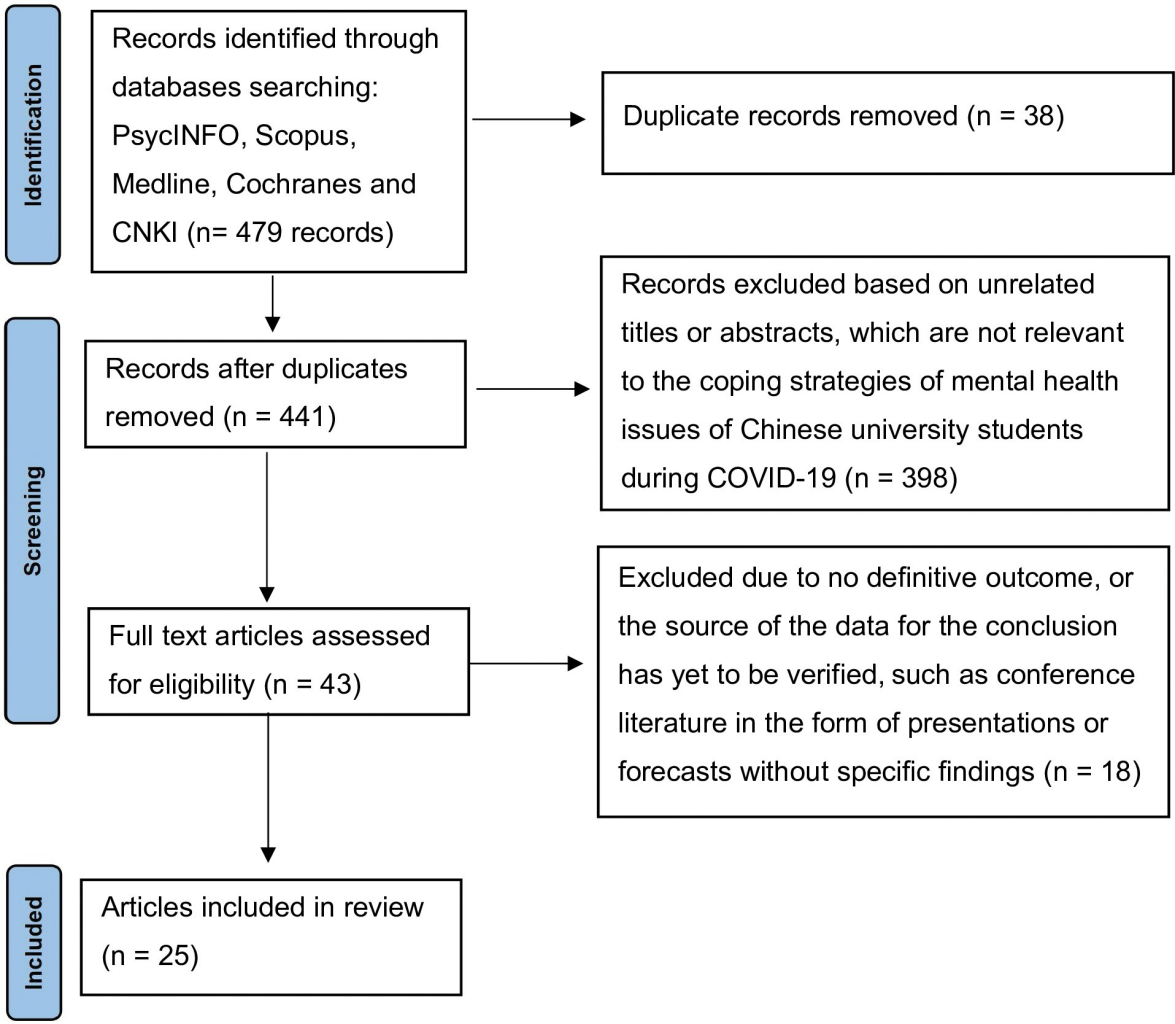
PRISMA flow diagram.

Following the search for literature, search results were screened. As shown in Fig 1, literature matching the criterion for screening and inclusion was included in this study. A PRISMA diagram was used to systematically document the search results, screening, and selection using the inclusion and exclusion criteria. Duplicate articles were removed, and titles and abstracts of the studies were evaluated for their applicability, and any article that was not relevant was excluded. This process avoided collecting repeated data to avoid bias in the outcomes [30]. In addition, the rapid review was conducted in accordance with the guidelines outlined in the PRISMA checklist (see S1 Checklist).

### Thematic analysis

Thematic analysis (TA), using six steps noted by Braun and Clarke, was used to identify patterns, organize them, and draw meaningful insights from the data [31]. During the first step, the researcher studied the textual material multiple times to develop familiarity with the data. Following this, initial codes were generated, and the data were analysed systematically by coding. In the third step, themes were generated from the code. This allowed for the analysis to take shape. In the fourth step, the themes were reviewed and refined. Some themes were expanded while others collapsed following the review. The themes were then named and defined, clearly stating what makes each topic unique and specific. During the final step, a report was generated based on the analysis. The use of this approach allowed for the minimization of biases in the analysis process. The themes of the review articles were coded and organized according to different categories.

## Results

The search yielded 479 articles: Scopus = 42, PsycINFO = 9, Cochrane = 10, Medline = 288, and CNKI = 130. In the initial screening, 38 duplicate articles were identified and removed. Subsequently, during the screening stage, 398 articles that did not contain the keywords relevant to this study in their titles or abstracts were excluded. In the full-text assessment for eligibility, we not only considered the relevance of the articles but also conducted a quality assessment. We assessed the quality of the studies based on various criteria, such as the rigor of their methodology, the reliability of their data sources, and the clarity of their reported outcomes. Articles that did not meet our quality criteria or had unspecific outcomes were excluded. After this comprehensive assessment, 25 articles were identified for inclusion in this study. All of these selected studies thoroughly examined the mental health of Chinese university students and their coping strategies during the pandemic time. The characteristics and main findings of these literature sources are detailed in Table 2.

**Table 2:** The characteristics and main findings of the literature reviewed.

Author, (Year)	Country	Study Design; Sample Size (N)	Summary of findings
Cao et al., (2020) [32]	China	Mixed qualitative and quantitative research N = 7143	Results indicated that 0.9% of the respondents were experiencing severe anxiety, 2.7% moderate anxiety, and 21.3% mild anxiety. Moreover, living in urban areas, family income stability and living with parents were protective factors against anxiety. Moreover, having relatives or acquaintances infected with COVID-19 was a risk factor for increasing the anxiety of college students.
Chen, S., (2021) [33]	China	Mixed qualitative and quantitative research N = 3373	Social support and positive coping styles were all negatively associated with loneliness. Social support directly affects loneliness, and coping styles mediate the effect of social support and loneliness.
Chinna et al., (2021) [34]	Malaysia, Saudi Arabia, Pakistan, Bangladesh, China, India and Indonesia	Cross-sectional study N = 3679	Females from Bangladesh, China, Malaysia, Pakistan and Saudi Arabia experienced significantly higher levels of anxiety compared to their male counterparts. Acceptance was the most used and Seeking Social Support was the least used coping strategies among the students.
Gan & Fu, (2022) [35]	China	Questionnaires and descriptive statistics N = 1038	The positive emotion or negative emotion of Chinese university students could have potential associations with specific coping behaviours.
Guo et al., (2022) [36]	China	Mixed qualitative and quantitative research N = 303	Mental cues and stress were important factors affecting mental health, and physical exercise could effectively regulate the mental health of individuals.
Huang et al., (2022) [37]	China	Cross-sectional study N = 35516	Junior-year undergraduates, postgraduates, only-child families, mental health history, community COVID-19 cases, depressive symptoms, and negative coping linked to suicidal thoughts. Conversely, higher social support, positive coping, and improved family function reduced the risk of lasting dysfunction amid lockdown.
Huang et al., (2021) [38]	China	Cross-sectional study N = 558	Active coping style and family support were protective factors while passive coping style could aggravate psychological problems among Chinese university students
Lin et al., (2020) [39]	China	Mixed qualitative and quantitative research N = 1315	Chinese university students have high levels of stress, low levels of anxiety and average coping skills; perceived stress is positively related to anxiety and negative coping, and negatively related to positive coping.
Li et al., (2022) [40]	China	Cross-sectional study N = 6027	The incidence of psychological distress was found to be 35.34%. Negative coping style and expressing panic about COVID-19 on social media were the most important predictors of psychological distress.
Li et al., (2021) [41]	China	Quantitative research N = 4355	COVID-19 particularly affected older male master’s and doctoral students living in Wuhan, especially for post-traumatic stress disorder.
Li & Peng, (2021) [42]	China	Cross-sectional study N = 2640	University students reported high levels of cognitive coping, behavioural coping, and social support. They also experienced low levels of anxiety and emotional coping. Anxiety was significantly and negatively related to coping and social support.
Li et al., (2021) [43]	China	Quantitative research N = 2640	The anxiety symptoms of Chinese university students were significantly and negatively associated with subjective support and counsellor support, while family support did not show a substantial association with anxiety.
Liao et al., (2021) [44]	China	Mixed qualitative and quantitative research N = 319	Six months after the COVID-19 outbreak, both Chinese international students and domestic university students showed moderate to high levels of PTSD, and positive and negative coping and social support had an impact on respondents’ PTSD symptoms, with social support being significantly associated with students’ choice of coping strategies.
Nurunnabi et al., (2020) [45]	China	Cross-sectional study N = 559	During the COVID-19 pandemic, Chinese university students experienced psychological concerns such as lack of sleep, emotional support, moral support and social attraction due to high levels of anxiety and psychological stress, they needed coping strategies and survival skills.
Sun et al., (2021) [46]	China	Cross-sectional study N = 1912	During the COVID-19 pandemic, psychiatric symptoms of traumatic stress, depression, anxiety and suicidal ideation were prevalent among Chinese university students. Financial stress, perceived COVID-19 social stigma and perceived COVID-19 threat were associated with higher symptom severity.
Tang et al., (2020) [47]	China	Cross-sectional study N = 2485	Feeling extreme fear was the most important risk factor for psychological distress among Chinese university students, followed by short sleep duration, impending graduation (fourth year) and living in a severely affected area. Sleep duration mediated the link between exposure and mental health problems.
Tian H., (2021) [48]	China	Mixed qualitative and quantitative research N = 472	Social work has creatively proposed practical pathways to solutions in the perspective of psychological prevention of university students. Psychological counselling, as a professional means of solving psychological problems in universities, played an important role in the social services of universities
Wang & Zhao, (2020) [49]	China	Mixed qualitative and quantitative research N = 3611	Chinese university students had higher levels of anxiety about the COVID-19 pandemic and there were significant differences between males and females.
Wang & Hu, (2021) [50]	China	Mixed qualitative and quantitative research N = 2636	The lowest levels of depression and anxiety among Chinese university students were found during the initial phase of effective control of the pandemic. University students from divorced parents and reconstituted families were at greater risk of depression. Ambivalence in the family environment was a common risk factor for mental health.
Wang & Yan, (2022) [51]	China	Longitudinal study N = 973	There was a longitudinal causal relationship between perceived risk of COVID-19 pandemic, physical activity and mental health, and male students had better physical activity and mental health than female students.
Ye et al., (2020) [52]	China	Cross-sectional study N = 7800	The direct and indirect effects of covid-19-related stressful experiences on ASD symptoms among Chinese university students were significant. Mental toughness, adaptive coping strategies and social support could mediate the relationship between stressful experiences associated with COVID-19 and ASD without significant moderation by maladaptive coping strategies.
Yang et al., (2020) [53]	China	Cross-sectional study N = 4139	The detection rates of depression and anxiety among university students were 39.0% and 26.9% respectively. There was a mediating effect of social support between virus exposure and depression anxiety scores.
Zhao et al., (2021) [10]	China	Cross-sectional study N = 666	Prevalence of depression among medical students was slightly lower during the COVID-19 pandemic, with coping methods mediating the relationship between mental toughness and depression.
Zhang et al., (2021) [54]	China	Cross-sectional study N = 5400	Depression and anxiety symptoms were experienced by university students during the college lockdown and were detected less frequently in males than in females.
Zhu & Hu, (2022) [55]	China	Cross-sectional study N = 180	Physical activity during the pandemic was effective in reducing negative emotions among university students, and there were no significant differences in the gender, type of major, or education level.

In Table 3, we summarized the main themes from the rapid review and their respective sub-themes. In the following section, we then explored each theme and its sub-themes in more detail.

**Table 3:** Main themes and sub-themes from the rapid review.

Main Themes	Sub-themes
	Anxiety
Mental health status	Post-traumatic stress disorder (PTSD) and acute stress disorder (ASD)
	Suicidal ideation and depression
	Social support
Coping strategies	Family support
	Negative and positive response

### Mental health status

Lockdowns and subsequent restrictions on movement and gathering during the COVID-19 period significantly impacted the mental health of Chinese university students. The literature revealed three broad impacts: (i) anxiety (ii) post-traumatic stress disorder (PTSD) and acute stress disorder (ASD) (iii) suicidal ideation and depression (see Table 4).

**Table 4:** Mental health issues among Chinese college students during the COVID-19 pandemic.

Subthemes	English literature	Chinese literature	Source
Anxiety	8	3	[32, 34, 38, 39, 42, 43, 45, 46, 48–50]
Post-traumatic stress disorder (PTSD) and acute stress disorder (ASD)	5	0	[43, 44, 46, 47, 52]
suicidal ideation and depression	5	1	[10, 37, 38, 46, 47, 54]

### Anxiety symptoms

Cao found that before implementing the Dynamic Zero COVID-19 policy in 2020, approximately 25% of university students expressed pandemic-related concerns, with most experiencing mild anxiety [32]. As COVID-19 cases increased and spread to more provinces, students’ anxiety levels heightened. Factors such as impact to parental income sources, living arrangements, and infected relatives were associated with increased anxiety levels [34, 38, 42, 43]. Control measures implemented during the spring semester delayed students’ return to campus, worsening their anxiety symptoms and increasing the proportion of affected students [45, 46]. Online courses further fuelled uncertainty about future education, increasing reports of sleep problems, nightmares, and stress levels [49]. This was further heightened by increased isolation and reduced social interactions [48]. Contrary to these findings, Lin reported that university students experienced less anxiety but higher perceived stress during isolation [39]. The disparity was more pronounced among students who were in the later stages of their studies, particularly medical students. Additionally, increased anxiety levels were reported in August 2020, coinciding with the normalization of pandemic prevention measures and causing uncertainty about students’ return to campus [50].

### Post-traumatic stress disorder (PTSD) and acute stress disorder (ASD)

When specific preventive measures were not implemented at the beginning stage of the pandemic, evidence suggests that less than 5% of college students had symptoms of PTSD [47, 52]. While long-term home isolation was not related to PTSD or depression, there was a direct correlation between stressors associated with the pandemic and elevated ASD symptom levels. Liao found that in August 2020, after the implementation of the dynamic zero policy, the PTSD level among Chinese university students increased to moderate to high-levels [44]. While PTSD symptoms at the early stages of the pandemic could be attributed to misdiagnoses and attributed to other stressors, the study demonstrated a direct impact of the dynamic zero policy on Chinese university students. Anxiety did not vanish in students; instead more symptoms associated with PTSD showed up over time. Similar results were noted about four months after the outbreak, indicating that COVID-19-infected university students were more likely to have PTSD compared to students who are COVID-19-negative [43, 46].

### Suicidal ideation and depression

In February 2020, following the strict lockdown measures, as much as 5% of university students had depression [47]. In March 2020, a follow-up survey revealed that nearly half of the university students had substantially worsened symptoms of depression [46]. This was a significant increase, and students reported extreme dread during the outbreak. The literature indicated a link between shorter sleep duration and poor mental outcomes. Those who resided in areas with high infection rates, and were in the later stages of their studies had the highest risk of developing depression. Interestingly, results indicated gender differences, which may be attributed to the pandemic prevention policies of the universities [10, 54]. Universities implemented specific measures and support systems for male and female students. Variations in these strategies and resources provided by universities may be linked to the differences in mental health outcomes for male and female students. Additionally, approximately one-fifth of university students reported having suicidal thoughts between March and April 2020. This was attributed to social and economic status, COVID-19-related family financial pressure, perceived social support, along with perceived COVID-19 danger [37, 38].

### Strategies for coping with the psychological impact

In response to the challenges posed by the COVID-19 pandemic, Chinese university students employed a number of coping strategies to minimise psychological impact. These strategies were categorized into three main themes, namely (i) social support, (ii) family support, and (iii) negative and positive response (see Table 5).

**Table 5:** Mental health coping strategies among Chinese university students during the COVID-19 pandemic.

Subthemes	English literature	Chinese literature	Source
Social support	4	2	[32–34, 42, 52, 53]
Family support	4	0	[37, 38, 41, 42]
Negative and positive response	4	3	[10, 36, 38, 43, 44, 51, 55]

### Social support

There is a negative correlation between the prevalence of anxiety and depression symptoms for university students and social support. The research consistently demonstrated that social support positively correlates with reducing anxiety levels [33, 42, 53]. Moreover, these studies reveal that social support was crucial in mediating the relationship between cognitive and behavioural coping strategies and anxiety. This confirmed the role of social support in reducing anxiety and highlights its function in promoting anxiety reduction. Cao found that during the pandemic social support mechanisms reduced [32]. However, when comparing coping mechanisms among students in China, Bangladesh, Malaysia, Saudi Arabia, and Pakistan, Chinese university students were found to have more possibilities to use social support-seeking skills, which may be attributed to the widespread application of social media in China [34]. Furthermore, it is worth noting that adaptability, social support, and resilience have been identified as robust factors providing individual and social protection against acute stress disorder (ASD), as outlined by Ye [52]. In the context of our results, it’s important to highlight that social media platforms have emerged as one of the avenues where individuals have demonstrated adaptability, social support, and resilience.

### Family support

Anxiety, depression, stress symptoms, and suicidal tendencies have substantial negative associations with family support [37, 38]. Family support has shown the potential to reduce mental illness severity, especially anxiety [42]. Moreover, perceived family support was an essential protective factor in preventing college students from experiencing negative mental health consequences and the exacerbation of suicidal tendencies during the pandemic. This was because family members are better positioned to communicate health needs, more inclined to support each other emotionally and have empathy for one another. In particular, benefits from family support arose from their ability to provide individualized care and support. This support appeared to demonstrate a more promising effect on lessening the severity of mental illness than help from a spouse or friend.

Clarifying further, Li suggested that family support and anxiety have an indirect relationship rather than a direct one [41]. This indirect relationship demonstrates that cognitive, emotional, and behavioural coping strategies could be helpful in mediating anxiety. The strong mediating effect suggests that living with family members may not be a key factor in reducing anxiety, but rather the adoption of appropriate coping strategies is necessary to activate the transmission of family support and reduce anxiety.

### Negative and positive response

Positive coping strategies clearly reduced the mental health impact on Chinese students as a result of lockdowns and other prevention and control measures, particularly depressive symptoms [10, 41]. This suggests that active coping strategies can support students to address challenges and problems more rationally and may alleviate stress, thereby preventing depression. During the pandemic, mental health issues and hazardous behaviours caused by alcohol and cigarette abuse emerged as a new health concern, particularly among undergraduate students. Using unhealthy coping strategies such as smoking and excessive drinking can harm the mental health of students. Individuals aged 14 to 35 commonly employed passive defense strategies, including emotional suppression and substance abuse. Interestingly, according to a survey of university students, nearly one-third of respondents admitted to utilizing negative coping strategies such as “smoking, drinking, taking drugs, or overeating to reduce worry” during COVID-19 [38]. Worryingly, this suggests that students with mental health issues such as stress and anxiety could adopt harmful behaviours as a coping mechanism. In contrast, adopting positive coping strategies could constructively regulate psychological distress and alleviate negative emotions. Moreover, female students employed positive coping strategies more than male students. This was because they had better emotional regulation, higher cognitive evaluation, and lower expression inhibition than male students [43]. The study further revealed that male students experienced greater psychological stress than female students.

During COVID-19, physical activity was identified as a positive coping strategy for Chinese college students to reduce their negative mental health effects [44]. Several studies investigated the connection between psychological health and physical exercise during this period. Most students reported that physical exercise helped them relax and manage psychological stress [36, 55]. This suggests that psychological health issues resulting from the COVID-19 pandemic or other crisis events can be alleviated by increasing awareness of physical exercise [51].

## Discussion

In this rapid review, we identified 25 articles related to the mental health status and coping strategies of Chinese university students during the COVID-19 pandemic. The review identified three areas of mental health impact for Chinese university students. These were: (i) anxiety (ii) post-traumatic stress disorder (PTSD) and acute stress disorder (ASD) (iii) suicidal ideation and depression. Moreover, three coping strategies that helped students alleviate the impacts of the pandemic on their mental health were also identified. These were: (i) social support (ii) family support (iii) negative and positive response.

### Mental health impacts and interventions

During the COVID-19 pandemic, Chinese university students experienced elevated anxiety levels, impacting their mental health [32, 42, 45, 49]. Despite variations in assessment methods, the findings consistently noted the substantial mental health impact of the pandemic on Chinese university students. Stressors such as financial constraints, distance learning, and uncertain future career prospects emerged as major contributors to anxiety among students [34]. Additionally, it was observed that female students exhibited higher anxiety levels, suggesting the need for further investigation into the underlying factors.

A significant number of Chinese university students also displayed symptoms of anxiety-related disorders such as ASD (Acute Stress Disorder) and PTSD (Post-Traumatic Stress Disorder) during the pandemic [43, 44]. International studies support these findings, indicating that university students globally were at risk of developing PTSD due to pandemic prevention and control measures [56, 57]. This emphasized the need for early screening tools to identify and address PTSD symptoms related to COVID-19. It is important to note that the gravity of the problem may be larger than reported in studies. Evidence suggests that early studies may have underestimated the prevalence of PTSD symptoms due to limited awareness and societal stigma surrounding mental health issues [58, 59]. As the pandemic progressed, the prevalence of PTSD increased, suggesting that ongoing mental health monitoring and interventions are crucial to address the long-term impact of the pandemic on university students’ mental well-being [46].

Moreover, the studies highlighted the role of intrinsic and interpersonal factors in mitigating the connection between COVID-19 stressors and elevated levels of ASD symptoms [52]. Factors such as social support, adaptive coping strategies, and resilience attenuated the impact of stressors on mental health outcomes. This underscores the importance of implementing effective coping strategies, emotional control techniques, and providing adequate social support to mitigate the mental health consequences experienced by Chinese university students.

Furthermore, the pandemic led to a notable increase in reports of depressive symptoms and suicidal tendencies among Chinese university students. Male students were more likely to develop depression symptoms, influenced by cultural and societal factors [60, 61]. The availability of social support and financial stress played important roles in exacerbating or mitigating the effects of the pandemic on mental health outcomes [32]. Additionally, sleep disturbances and shorter sleep duration were reported by students during the pandemic and were associated with negative psychological consequences, including depressive symptoms [62]. Understanding the bidirectional relationship between mental health and sleep, addressing cultural factors, providing social support, and alleviating financial stress are essential in promoting the well-being of Chinese university students in the context of the pandemic.

The cumulative impact of the pandemic on the mental health of Chinese university students highlights the need for comprehensive interventions. This includes addressing social and economic inequalities, promoting healthy sleep habits, and ensuring equitable access to mental health care. Efforts should focus on providing necessary resources and support, particularly for vulnerable populations from lower socioeconomic backgrounds. By strengthening mental health support systems and taking proactive measures, universities and policymakers can create an environment that prioritizes the well-being of university students, fostering resilience in the face of future challenges.

### Coping strategies and social support

The mental health of Chinese university students during the COVID-19 pandemic was influenced by various factors, including social support, family support, and coping strategies. Social support played a crucial role in supporting the mental well-being of these students. There are strong links between social support and the prevalence of anxiety and depressive symptoms, where poor support systems have increased prevalence [32, 33, 63]. By reducing psychological stress, social support effectively mitigates depression and anxiety among university students. This highlights the importance of fostering social support and creating a supportive community within educational institutions.

Interestingly, Chinese university students preferred to seek social support online, utilizing platforms such as WeChat, QQ, and other social media platforms [64]. This suggests that social media can provide a convenient and accessible way for students to seek support during times of crisis. Leveraging technology and social media in supporting students is an important understanding, and can have benefits beyond the Chinese student population. It is therefore important to raise awareness of and potential benefits of leveraging social media and technology to emulate support mechanisms during times of crisis. By utilizing social media platforms, educational institutions can create online communities that foster connection and facilitate open discussions about mental health and combating feelings of isolation. Social support exhibits a strong association with anxiety reduction, making it vital for institutions to emphasize its importance and provide guidance on using technology to access support networks [59, 65]. Through targeted campaigns and resources, universities can create a supportive online environment encouraging students to connect with peers, seek help, and reduce anxiety levels.

Family support has been found to impact the psychological health of Chinese university students during the pandemic. Specifically, perceived family support protects against negative mental health consequences [38, 42]. In particular, family support and good functioning reduces feelings of loneliness and anxiety among students and safeguards against the exacerbation of suicidal tendencies [37, 41]. Good family functioning is characterized by a strong adaptation to the pandemic, supportive communication and good social connections. Family ties and support are important in Chinese culture, where filial piety, the Confucian principle of respecting and caring for one’s parents and ancestors, is considered a fundamental virtue [66]. The strong emphasis on family relationships and intergenerational support in Chinese society reflects the importance of filial piety, which has significant implications for social support function in Chinese university students’ mental health.

However, while family support is important, addressing mental health issues during the pandemic may not be sufficient. Appropriate coping strategies are necessary to activate the transmission of family support and decrease anxiety levels. Effective coping strategies, including cognitive, emotional, and behavioural approaches, can reduce anxiety levels and increase resilience. Cognitive strategies involve reframing negative thoughts and adopting more adaptive thinking patterns, while emotional strategies involve managing and expressing emotions in healthy ways [67]. Behavioural strategies focus on taking proactive actions, such as engaging in physical exercise, practicing relaxation techniques, or seeking professional help when needed [36]. Educational institutions and mental health professionals should focus on promoting cultural connections to family, tapping into family connections and support to equip students with effective coping strategies to manage stress and reduce anxiety. As a result, many educational institutions have launched mental health awareness campaigns to destigmatize mental health issues and encourage students to seek family support [68]. These campaigns aim to educate students, faculty, and staff about the importance of family support and coping strategies in managing stress and anxiety. Implementing support services such as family counselling, peer support, and mentoring programs can also strengthen family relationships and create a more supportive community [69].

Positive coping strategies were found benefit Chinese university students’ mental health, while negative coping mechanisms were associated with hazardous behaviours and worsened mental health outcomes [10]. Active coping strategies, such as problem-solving and stress reduction, enhanced mental health outcomes more effectively than avoidant coping. Students with positive coping strategies dealt with problems more rationally, reduced stress, and prevented depression. However, some students resorted to negative coping strategies such as substance abuse or overeating to alleviate worry, leading to harmful behaviours and worsened mental health outcomes [38].

Seeking social and familial support has been associated with lower reactions to post-traumatic stress disorder (PTSD) symptoms. Numerous studies have shown that having a strong support system from family members and peers can mitigate the negative impacts of traumatic experiences on mental health outcomes [70, 71]. This highlights the importance of promoting social connections and fostering supportive relationships for individuals experiencing PTSD symptoms, including Chinese university students during the pandemic and future crisis events. Physical activity has also been recognized as an effective coping mechanism for reducing negative mental health effects among university students. Engaging in regular exercise has been associated with improved psychological well-being, stress reduction, and increased resilience [72, 73]. The positive effects of physical exercise can be attributed to its ability to help individuals relax, release tension, and cope with psychological stress. Notably, physical exercise has been found to exhibit a negative correlation with stress levels, emphasizing its potential as a valuable coping strategy for Chinese university students facing mental health challenges during the pandemic [36, 51, 55].

However, it is crucial to acknowledge the presence of gender differences in coping strategies and psychological stress levels. Evidence indicates that women tend to exhibit higher positive coping strategies, including seeking social support and engaging in emotional expression [74, 75]. Conversely, men often report experiencing greater psychological stress and may rely on alternative coping mechanisms, such as avoidance or substance use [76, 77]. These gender differences underscore the need for gender-specific support and intervention strategies to address the unique challenges faced by female students and promote their mental well-being.

### Strengths and limitations

In this rapid review, we acknowledged both its strengths and limitations. The strengths of this study were rooted in its timeliness and adaptability. As the research focused on the rapidly evolving COVID-19 pandemic’s impact on Chinese university students, a rapid review was well-suited for efficiently and cost-effectively investigating limited literature. The study’s potential to bridge knowledge gaps, inform policy and practice, and offer timely insights into the mental health of this population added to its relevance and impact. However, some limitations to this review should be noted. First, due to the rapid nature of this review, we may have missed some relevant studies that were not included in our search. Additionally, the studies’ reviewed varied in quality and there were some inconsistencies in the methods used to measure mental health and coping strategies. This could impact the accuracy of our findings and conclusions. Finally, the review focused solely on Chinese university students and may not be generalisable to other populations or settings, given the uniqueness of the Chinese dynamic zero policy.

Although limitations still existed, the review formulated a useful summary of the current research on the coping strategies and mental health of Chinese university students. By synthesizing the existing research, it was possible to identify research gaps and devise targeted interventions to support the mental health of students during and after the pandemic.

## Conclusion

In conclusion, this rapid review highlights the significant impact of COVID-19 on the mental health of Chinese university students. The pandemic has increased anxiety, depression, stress, and social isolation among students. Positive coping strategies highlighted the mitigating benefits of seeking social and family support and engaging in positive activities like physical exercise on mental health. However, the existing literature has limitations in terms of study availability and standardized measurements. Nonetheless, this review provides valuable insights into the mental health needs of Chinese university students during the pandemic and emphasizes the importance of providing resources and ongoing support. Lessons from the experiences of Chinese students during the pandemic provide opportunities for Government health authorities and educational institutions to strengthen coping strategies for student population groups in response to future crisis events. Government health authorities should formulate policies prioritising students’ well-being and implementing measures to support their mental health. This includes establishing mental health support services within universities, conducting regular monitoring, and reducing the stigma associated with seeking help. Education institutions should incorporate coping strategies into the curriculum and provide educators training on mental health to be able to better respond. Additionally, the role of the family is central to mitigating the impact on mental health. Both government health authorities and educational institutions should actively engage families in the design of support services and curricula on mental health and effective coping strategies. Collaboration between health professionals, educators and family is essential for a coordinated approach, multi-sector approach. By implementing these recommendations, multisectoral support can be provided to foster resilience and mitigate the negative impacts on students’ mental health during and beyond the pandemic.

## Supporting information

S1 Checklist (DOCX)
